# The Rat Medial Prefrontal Cortex Exhibits Flexible Neural Activity States during the Performance of an Odor Span Task

**DOI:** 10.1523/ENEURO.0424-18.2019

**Published:** 2019-03-26

**Authors:** Emanuela De Falco, Lei An, Ninglei Sun, Andrew J. Roebuck, Quentin Greba, Christopher C. Lapish, John G. Howland

**Affiliations:** 1 Department of Psychology, Indiana University-Purdue University Indianapolis, Indiana 46202; 2 Department of Anatomy, Physiology, and Pharmacology, University of Saskatchewan, Saskatoon, Saskatchewan S7N 5E5, Canada

**Keywords:** electrophysiology, multivariate statistics, odor, prefrontal cortex, pyramidal neuron, working memory

## Abstract

Medial prefrontal cortex (mPFC) activity is fundamental for working memory (WM), attention, and behavioral inhibition; however, a comprehensive understanding of the neural computations underlying these processes is still forthcoming. Toward this goal, neural recordings were obtained from the mPFC of awake, behaving rats performing an odor span task of WM capacity. Neural populations were observed to encode distinct task epochs and the transitions between epochs were accompanied by abrupt shifts in neural activity patterns. Putative pyramidal neuron activity increased earlier in the delay for sessions where rats achieved higher spans. Furthermore, increased putative interneuron activity was only observed at the termination of the delay thus indicating that local processing in inhibitory networks was a unique feature to initiate foraging. During foraging, changes in neural activity patterns associated with the approach to a novel odor, but not familiar odors, were robust. Collectively, these data suggest that distinct mPFC activity states underlie the delay, foraging, and reward epochs of the odor span task. Transitions between these states likely enables adaptive behavior in dynamic environments that place strong demands on the substrates of working memory.

## Significance Statement

Working memory capacity is altered in psychiatric disorders including schizophrenia. In the present work, we describe activity states of neurons in medial prefrontal cortex as well trained rats perform the odor span task, which has been nominated as a task suitable for studying working memory capacity in rodents. Our results demonstrate dynamic and flexible activity patters during different epochs of the task (i.e., the delay period, foraging, reward collection, and after making errors). Our results bolster contemporary theories of the medial prefrontal cortex exhibiting metastable dynamics in complex environments. Disruptions in these dynamics may underlie some cognitive symptoms of schizophrenia, including reduced working memory capacity.

## Introduction

Working memory (WM) refers to the ability to hold and manipulate information online during a delay for future use. Understanding the neurobiological bases of WM is critical as deficits in WM are a central cognitive symptom of brain disorders including schizophrenia (Barch and Smith, 2008; Barch et al., 2012). WM can be broadly parsed into domains including goal maintenance, interference control, and capacity (Barch and Smith, 2008; Barch et al., 2012). Each of these domains requires several cognitive functions, such as planning, executive control, resistance to distraction, task monitoring, and memory. Electrophysiological recordings performed in animals engaged in WM tasks have identified the types of computations that brain regions or networks contribute to these functions (Constantinidis et al., 2018; Lundqvist et al., 2018). Prior work indicates that optimal working memory performance is accompanied by ensembles of medial prefrontal cortex (mPFC) neurons that track the various requirements of a task (e.g., epochs, rules; Lapish et al., 2008, 2015; Durstewitz et al., 2010; Del Arco et al., 2017), which may facilitate performance monitoring and error detection in these networks (Hyman et al., 2017). Trial-specific information is also thought to be maintained over a delay for optimal performance of WM tasks. The role of the mPFC in the maintenance of information across a delay has been extensively interrogated and two hypotheses have emerged. Initially, the identification of neurons that are persistently active during the delay suggested that mPFC may serve as a “buffer” to temporarily hold information (Goldman-Rakic 1996; Funahashi 2015). However, this view has evolved to suggest that mPFC is more important for directing cognitive resources/attention toward relevant neural circuits that likely play a defined role in the stimulus maintenance (Curtis and D’Esposito, 2003; Postle 2006; Tsujimoto and Postle, 2012; Lara and Wallis, 2015). For example, we found that dynamic changes in theta power (mPFC and hippocampus) and increased mPFC unit phase locking to hippocampal theta during the delay period of a spatial WM task predicted subsequent performance during the test phase of the spatial WM task (Myroshnychenko et al., 2017). Therefore, the goal of this study was to further understand the contributions of the rodent mPFC to WM by measuring neural activity in a validated measure of WM, the odor span task.

**Figure 1.**
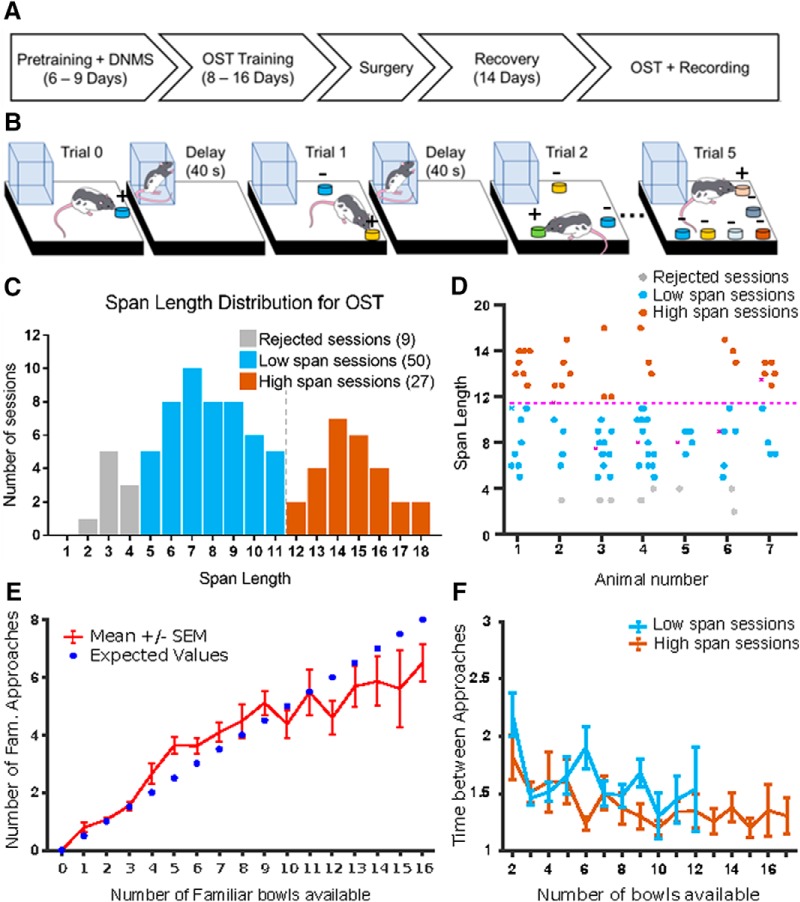
***A***, Timeline depicting experimental events. Pretraining and DNMS required 6–9 d of training. Training on the OST required 8–16 d of training. Following OST, animals underwent electrode implantation surgery and were allowed 14 d to recover. Following recovery, OST resumed, and electrophysiological recording occurred. ***B***, OST consists of successive trials in which the animal must identify a novel odor and dig to receive a food reward. Different colors indicate different odors. With each successive trial, a new odor bowl (+) is added, while the previous odors (−) are rearranged pseudorandomly. Between each trial the animal returns to a clear Plexiglas house for an intertrial delay period of ∼40 s. OST continues until the animals fails to dig in the novel bowl. Span length is determined as the number trials successfully completed. ***C***, Distribution of span lengths across the 86 recording sessions. The distribution is not unimodal (Calibrated Hartigan’s dip test, *D*_(86)_ = 0.048, *p* = 7.2 × 10^−3^). The local minimum between the two peaks (span = 11.5, black dotted line) was taken as threshold to classify the sessions into Low span (blue) and High span (red). Nine sessions with a span length smaller than five were excluded from the following analysis (grey). ***D***, Span length for each session plotted by individual rats. Most rats (6/7) had both low and high span sessions (ANOVA test, *F*_(6,79)_ = 1.78, *p* = 0.11). ***E***, Average number (±SEM) of familiar bowl approaches versus number of familiar bowls available (red). The numbers of bowls visited prior to a correct dig was compatible with the statistically expected ones (blue dots; FDR-corrected *t* test, *p* > 0.05 for all spans). ***F***, Average time (±SEM) between approaches versus number of bowls available in High and Low span sessions. No difference was found for any number of bowls between 2 and 12 false discovery rate (FDR-corrected *t* test, *p* > 0.05).

Recently, the NIH-initiated Cognitive Neuroscience Treatment Research to Improve Cognition in Schizophrenia group nominated the odor span task (OST) for assessing WM capacity in rodents (Dudchenko et al., 2000, 2013; Young et al., 2007). The OST is an incremental nonmatching-to-sample task that closely resembles human working memory tasks that assess span, a rare characteristic for rodent WM tasks (Dudchenko et al., 2013). While performing the OST, rodents are required to dig for food rewards in scented bowls (Fig. 1*B*) and typically achieve spans of 8 to 15 odors in the task, although higher spans can be attained under some conditions. Performance of the OST depends on a distributed neural circuit including mPFC and dorsomedial striatum but not the hippocampus or posterior parietal cortex (Dudchenko et al., 2000; Davies et al., 2013a,b; 2017; Scott et al., 2018). Span is also impaired by manipulations related to schizophrenia including treatment with NMDA receptor antagonists (Rushforth et al., 2011; Davies et al., 2013a, 2017; Galizio et al., 2013) and maternal immune activation during pregnancy (Murray et al., 2017). However, to date, no studies have assessed the patterns of neural activity underlying OST performance.

Given the critical role of mPFC in the OST and the task’s similarity to human span tasks, we measured patterns of mPFC neural activity during performance of the OST in well trained rats. To identify changes in neural activity required for optimal performance of the task, high span versus low spans sessions were analyzed during three task epochs: (1) the delay period, (2) the foraging period, and (3) the reward (or error) period. We predicted that delay-period activity of mPFC neurons recorded during the OST would predict span length in the OST. During foraging in the OST, rats approach familiar and novel bowls and sample the odors in exactly the same manner; however, when a novel odor is detected, they dig to retrieve the food reward. Thus, inhibition of digging is critical for performance of the task, particularly as the number of stimuli on the platform increases and the rats visit more familiar bowls during a bout of foraging. As proactive inhibition of motor responses is a recently described function of mPFC (prelimbic region; Hardung et al., 2017), we anticipated to detect an inhibition signal during the foraging period. Finally, previous studies in decision-making tasks have shown error-related signals in mPFC neurons (Totah et al., 2009; Bissonette and Roesch, 2015; Laubach et al., 2015), suggesting that this area is important for monitoring the outcome of actions during behavior. During the reward epoch of the OST, digging in the novel bowl enables retrieval of reward whereas the identical response (i.e., digging) in a familiar bowl is an error and results in the end of the session. Thus, by directly compared neural activity in these two types of trials, we were able to generate a pure error signal. To the best of our knowledge, no other task allows for a direct assessment of WM span or capacity in this manner. Thus, our results will inform theories of mPFC function as the maximal working memory load (or capacity) is reached.

## Materials and Methods

### Subjects

Seven adult male Long–Evans rats (220–250 g at arrival, Charles River Laboratories) were used. Rats were paired housed for 1 week in standard ventilated plastic cages on a 12 h light/dark cycle (lights on at 07:00). Food and water were available *ad libitum*. Rats were switched to individual cages, food restricted, and handled for ∼5 min per day for 5 d before training commenced. Body weight was maintained at 85–90% of free-feeding weight throughout the behavioral tasks. All experiments were performed according to the Canadian Council on Animal Care and were approved by the University of Saskatchewan Animal Research Ethics Board.

### Behavioral apparatus

Training and testing occurred on a 91.5 cm^2^ black corrugated plastic platform with a 2.5 cm tall border. The platform was fastened to a metal frame with casters attached and stood 95 cm above the ﬂoor. It was surrounded by a beige curtain to block visual cues in the testing room. A Plexiglas box with a swinging door was placed in one corner of platform. Rats began each session in the box and were trained to go back to the box after obtaining reward for the delay period. The door was opened when trials started and closed when rats ran back into the box. Pieces of Velcro were equally spaced along the edge of the platform and used to fasten the sand ﬁlled bowls to the platform and stop the rats from spilling the sand. The bowls for a given trial were placed randomly on the pieces of Velcro. Odors were mixed in Premium Play Sand (Quikrete Cement and Concrete Products) and then placed in white porcelain bowls (4.5 cm high, 9 cm in diameter) on the platform as needed for each trial. Sand (100 g) was scented by mixing 0.5 g of a single dried spice purchased from a local grocery store allspice, anise seed, basil, caraway, celery seed, cinnamon, cloves (0.1 g), cocoa, coffee, cumin, dill, fennel seed, garlic, ginger, lemon and herb, marjoram, mustard powder, nutmeg, onion powder, orange, oregano, paprika, sage, and thyme. The order of the odors used each day was selected randomly and rats were exposed to all odors many times before recordings began.

### Pre-surgery training

Dig training: rats were trained to dig for a food reward (Kellogg’s Froot Loop) in a bowl filled with 100 g of unscented sand. Rats were placed opposite to a bowl on the platform for three separate phases. In the first phase, the food reward was positioned on top of the sand, in the second phase the food reward was incompletely buried, and in the third phase, the food reward was fully buried in the sand. Rats were trained until they would consistently dig for the food reward regardless of bowl position on the platform. This phase of training took 6–9 d to complete.

Delayed non-matching-to-sample (DNMS) task: in the sample phase (Trial 0), the rat was presented with a bowl of scented sand randomly positioned on the platform. Once the rat retrieved the Froot Loop, it was gently guided back to the box for the delay (40 s). In the choice phase of the trial (Trial 1), the previously presented bowl was randomly repositioned and a second bowl with a different odor was placed on the platform. A correct choice to obtain reward was recorded when rats dug into the bowl containing the novel odor. Rats moved on to the odor span task when they made 5 correct responses in 6 trials for 3 d.

OST: trials of the OST (Fig. 1*B*) were run as described for DNMS task except that bowls with novel odors were added in subsequent trials until rats made an error (i.e., dug in any of the bowls except the novel one). The delay was maintained at 40 s. Aspects of the protocol were designed to minimize the influence of extraneous factors on task performance and neuronal activity. Once the rat was in the holding box, bowls for the subsequent trial were positioned on the platform out of the view of the rat. To prevent spatial cues from influencing performance, previous bowls were randomly positioned for each subsequent trial. Once rats achieved a span of seven for 2 training days (8–16 d of training), the electrode array was implanted.

### Electrode implantation

Probes were custom built and consisted of a 4 × 8 matrix of tungsten wires (25 µm, California Fine Wire) in 35 Ga silica tubing (World Precision Instruments). They were then attached via gold pins to an EIB-36-PTB board (NeuraLynx). Impedance to 200–600 kΩ measured at 1 kHz (NanoZ, White Matter). Before surgery, rats were anesthetized with isoflurane, placed in a stereotaxic apparatus, and the dorsal surface of the skull was exposed. Four or five jeweler’s screws were threaded into the skull. Electrode arrays were then slowly lowered into medial prefrontal cortex (AP + 3.5–3.8 mm, ML ± 0.5 mm to the bregma, DV −3.5 mm from the dorsal surface of the brain). A stainless-steel wire served as the ground and was soldered onto one of the skull screws dorsal to the cerebellum. Dental acrylic was used to secure the electrode array to the skull and screws. Rats were treated with Anafen immediately following surgery and allowed to recover for 14 d before being retrained on the OST.

### Electrophysiological recordings

Rats were retrained on the OST until their spans were >4 for 2 d in row. Once this criterion was met, electrophysiological recordings were initiated during daily OST sessions. Rats were connected to a Digitalynx recording system controlled by Cheetah acquisition software (NeuraLynx). Unit signals were recorded via a HS-36 unit gain headstage mounted on animal’s head by means of lightweight cabling that passed through a commutator (NeuraLynx). Unit activity was amplified, sampled at 32 kHz, and bandpass filtered at 600–6000 Hz. Local field potentials were sampled at 32 kHz and filtered at 0.1–9000 Hz from each electrode. To verify the stability of recording, unit activity was recorded for ∼15 min before and after the behavioral session. Behavior of the rats during the OST was monitored by a camera mounted to the ceiling with the experimental time superimposed on the video for off-line analysis by a trained observer. Timestamps corresponding to trial start (when hindpaws exited the Plexiglas box), delay start (re-entry to the Plexiglas box), familiar approach, novel approach, dig (forepaws contacting the sand), reward, and errors were recorded for each OST session.

### Histological verification of electrode positions

After the completion of all recording sessions, rats were deeply anesthetized with isoflurane, electrode positions were marked by electrolytic lesions (10 μA current for 10 s), and then the rats were perfused transcardially with physiological saline followed by 10% formalin. Brains were removed and postfixed in a 10% formalin-10% sucrose solution. Brains were sectioned on a sliding microtome and infusion sites were determined using standard protocols with reference to a rat brain atlas (Paxinos and Watson, 2006).

### Unit isolation and criteria

Spike sorting was performed offline with SpikeSort 3D, using a combination of KlustaKwik and manual procedures. Multiple parameters (spike height, trough, and energy) were used to visualize the clustered waveforms. Each cluster was then checked manually to ensure that the cluster boundaries were well separated, and waveform shapes were consistent with action potentials. Refractory period violations, which were identified as spikes with an interspike interval of <1 ms, were also assessed. Using this criterion, none of the cells in the data set contained more than 0.9% of violations, and 22.2% of cells contained between 0.1 and 0.9% of violations. As electrodes were fixed, it must be acknowledged that units come from overlapping populations and individual units may have been sampled during more than one span session.

### Data analysis

Data analyses were conducted with custom routines in MATLAB (MathWorks).

Number of familiar bowls approached: as the number of bowls available increases, we expected the total number of bowls visited per trial to increase too. To verify this, we counted, on each trial, the number of familiar bowls approached prior to a correct dig and compared it with the statistically expected number of explorations (Fig. 1*E*), obtained as follows. Given *N* bowls of which only 1 is novel, and hypothesizing that once a bowl has been explored, the rat would not come back to it (no replacement), the expected number of explorations before finding the correct bowl is the sum of possible exploration numbers weighted by their probabilities:

$$E\left(N\right)=\sum_{i=1}^{N-1}i\cdot {Pf}_{i-1}\cdot {Pc}_{i},$$

where *P*f*_i_* is the probability of having failed all the explorations up the *i*th, and Pc*_i_* is the probability of finding the correct bowl on the *i*th exploration. Note that the sum stops at N − 1 because we are only counting the explorations prior to the correct dig. By substituting the probabilities in the equation, the expected number of explorations can be written as follows:

$$E\left(N\right)=\overset{N-1}{\underset{i=1}{\sum }}i\cdot \frac{N-\left(i-1\right)}{N}\cdot \frac{1}{N+1-i}=\overset{N-1}{\underset{i=1}{\sum }}\frac{i}{N}=\frac{N-1}{2},$$

where we used the known sum of the series of natural numbers in the last step.

**Figure 2.**
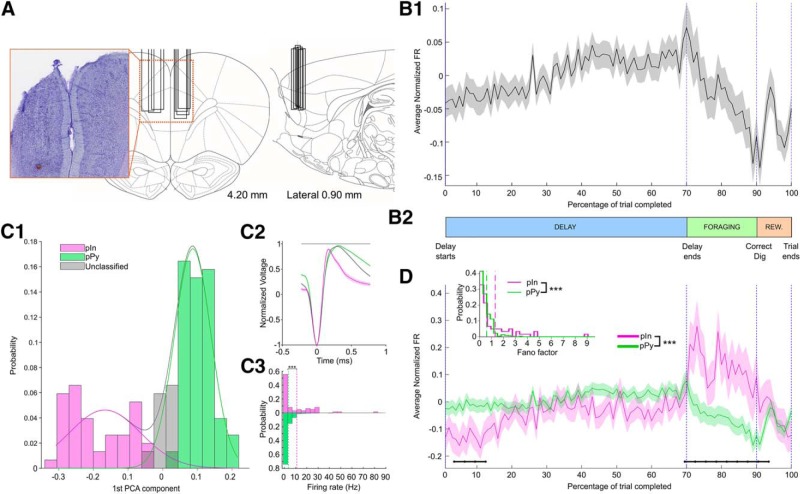
Task-normalized firing rates for pyramidal cells and interneurons. ***A***, ***D***, Coronal and sagittal rat brain sections depicting the location of the recording sites and photograph of a representative electrode placement. Probes were located in the prelimbic region of the mPFC. Box indicate the medial-lateral (left) and anterior–posterior (right) locations of the electrode arrays. ***B1***, Grand-average (±SEM) of task-normalized firing rate for 382 neurons recorded across 77 recording sessions. Firing rates were *z*-scored before averaging across neurons. ***B2***, Timeline of a single trial, where the three main epochs of the task (Delay, Foraging, and Reward) were identified through the four behavioral timestamps: Delay starts; Delay ends; Correct dig; and End of trial. Specific percentages of completion were assigned to each task epoch to calculate the task-normalized firing rates (see Data analysis – Task normalized firing rates). ***C1***, Distribution of first PCA components (integrating two waveform features) for pIn, pPy, and unclassified neurons. The Gaussian fits used for the classification are shown as continuous lines on top of the distribution. ***C2***, Mean waveforms (±SEM) for the three classes of neurons detailed in ***C1***. Unclassified neurons had a mean waveform closer to the pPy class and where subsequently labeled as pPys. ***C3***, Distribution of mean firing rates for 61 pIns and 321 pPy. Firing rates were higher in the pIn population than in the pPy one (Kolmogorov–Smirnov test, *D*_(321,61)_ = 0.30, *p* = 1.1 × 10^−4^). Vertical dotted lines mark the mean value of each distribution. ***D***, Grand-average (±SEM) of task-normalized firing rate for pIns and pPys. The firing rates in the two classes were significantly different (two-way ANOVA, interaction cell class × time, *F*_(99,38000)_ = 3.02, *p* = 8.4 × 10^−22^). Black horizontal lines mark groups of time bins with significant differences between pIns and pPy (FDR-corrected rank-sum, *p* < 0.05). Top Left, Distribution of Fano factors for pPys versus pIns (dotted lines mark mean values). Pins exhibit higher trial-to- trial variability (Kolmogorov–Smirnov test, *D*_(321,61)_ = 0.30, *p* = 9.8 × 10^−5^). ***p < 0.001.

Task normalized firing rates: for each correct trial, the three main epochs of the task (Delay, Foraging, and Reward epoch) were identified through the four behavioral timestamps: Delay start; Delay end; Correct dig; and End of trial (the latter corresponding to the Delay start marker of the following trial). A fixed percentage of trial completion was associated to each epoch by assigning a specific number of time bins (*N*) to it. Specifically, 70 time bins were assigned to the delay epochs, 20 to the foraging epochs, and 10 to reward epochs, for a total of 100 bins (Fig. 2*B2*). The spike trains in each trial occurring between the beginning (T*_j_*_1_) and end time (T*_j_*_2_) of a specific epoch *j* were binned into *N_j_* time intervals of duration *t_j_*:

$$t_{j}=\frac{T_{j2}-T_{j1}}{N_{j}}.$$

For a given neuron *i*, the firing rate in hertz along epoch *j* was obtained by dividing its corresponding bin counts for the time interval *t_j_*:

$${FR}_{ij}=\frac{{BinCounts}_{ij}}{t_{j}}.$$

The single neuron’s firing rates across all epochs and trials were then *z*-score normalized. Finally, for each neuron, a task-normalized firing trace describing its mean activity during the execution of the task was obtained by averaging across trials. The task-normalized firing rates on the error trials were obtained following a similar procedure, replacing the Correct Dig marker with the Error Dig one, and setting the End of Trial marker at 10 s after the error dig. A separate analysis was performed using a fixed binning procedure to ensure that the results reported herein were not attributable to the task-normalized binning procedure (data not shown). Comparisons between the fixed and task normalized binning procedures lead to identical conclusions.

Waveform-based classification putative interneurons and pyramidal neurons: to separate putative interneurons from pyramidal neurons, single units were classified based on their average action potential (AP) waveforms using a clustering protocol proposed by Ardid et al. (2015). For each of the 382 single units considered, action potentials were averaged and normalized in amplitude between −1 and 1. Each average waveform was interpolated with a cubical spline (from 32 original samples to 320 interpolated samples over 1 ms time interval). Two features of the resultant waveform were then measured: the peak-to-trough duration and the time for repolarization (time, after the peak, to reach 25% of peak amplitude). Using principal component analysis (PCA), we integrated these two features into the first principal component (explaining 84% of total variance). The distribution of first components was tested for bimodality using a calibrated Hartigan’s dip test (Cheng and Hall, 1998; *D*_(152)_ = 0.036, *p* = 6.2 × 10^−3^). We fit the distribution with two Gaussian models and defined cutoffs to separate the two groups of narrow and broad waveforms (Fig. 2*C1*). The two cutoffs were defined as the points at which the likelihood to belong to a group was 10 times larger than the likelihood to belong to the other one. Neurons with a principal component value smaller than the first cutoff (narrow waveforms) were classified as putative interneurons (pIn), whereas neurons with values larger than the second cutoff (broad waveforms) were classified as putative pyramidal cells (pPy). Neurons with a first component value falling between the two cutoffs were initially left unclassified. In several cases the AP waveform did not reach the repolarization threshold within the number of samples stored. Those cells were subsequently classified based only on their peak-to-trough duration which was compared with the peak-to-trough distributions for the classified waveforms (the peak-to-trough value had to exceed 5% confidence interval of the class distribution to be included in that class). Based on their average waveform (Fig. 2*C2*), the initially unclassified cells were subsequently merged with the pPy group. When looking at the firing rates of cells in each of the two classes (Fig. 2*C3*,*D*), we found that, as expected, the pIn population exhibited higher firing rates than the pPy one (Kolmogorov–Smirnov test, *D*_(321,61)_ = 0.30, *p* = 1.1 × 10^−4^), and higher Fano factors (Kolmogorov–Smirnov test, *D*_(321,61)_ = 0.30, *p* = 9.8 × 10^−5^).

The task-normalized firing rates for pIns and pPys were compared through a two-way ANOVA, where the interaction of cell class (pIn or pPY) and percentage of task completed (bins spacing from 1 to 100) was tested (interaction cell class × time, *F*_(99,38000)_ = 3.02, *p* = 8.4 × 10^−22^). The firing rate between pIns and pPys at specific times were compared by re-binning the time-normalized data in 33 bins and differences in a given time bin were detected via FDR-corrected rank sum tests (Fig. *2D*).

**Figure 3.**
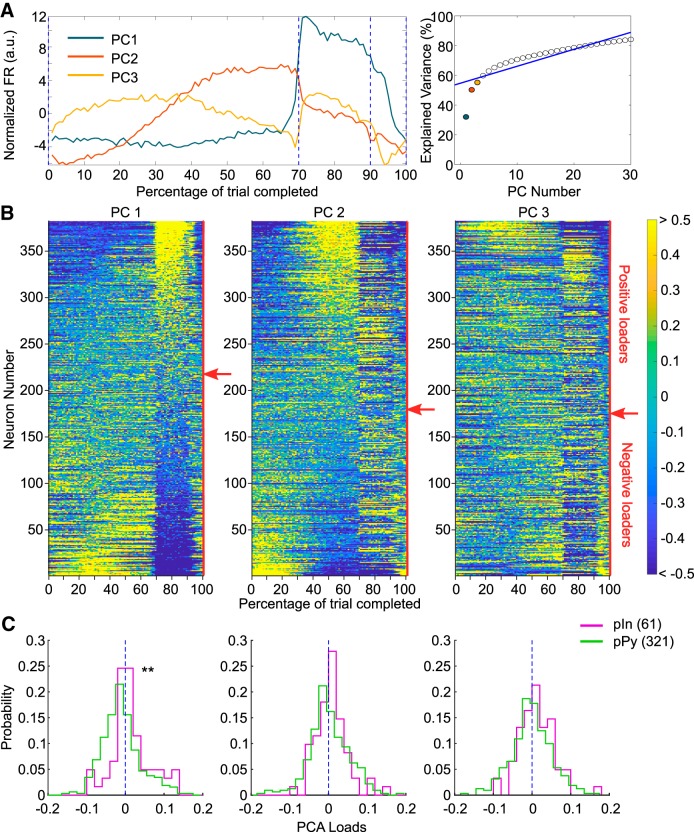
Identification of neural populations via PCA. ***A***, First three PCs. Projection of firing rates for the 382 neurons along the first three principal eigenvectors identified through PCA (left) and variance explained by each PC (right; blue line marks the broken stick model fit on the data). The first three PCs together explained 56% of the original variance of the dataset. ***B***, Task-normalized firing rates for the 382 neurons identified sorted according to their loadings on first, second, and third PC (left, center, and right, respectively). Red arrows on the right side of each color-plot indicates the transition point between positive and negative loaders. ***C***, Distributions of loadings on each PC separated for pIns and pPys. On the first PC pIns’ loadings were significantly higher than pPys’ ones (left; Kolmogorov–Smirnov test: *D*_(321,61)_ = 0.24, *p* = 4.9 × 10^−3^), whereas no significant effect was found on the other two PCs (Kolmogorov–Smirnov test: *D*_(321,61)_ = 0.10, *p* = 0.63 for PC2; *D*_(321,61)_ = 0.12, *p* = 0.37). **p < 0.01.

Identification of neural activity patterns via PCA: a principal component analysis was performed on the matrix of mean firing rates (*F*). Each column of the matrix *F* (100 × 382) contained the firing rate of a single neuron across the 100 time bins defining a trial of the task. We considered the first three principal components (PC) obtained, which, together, explained 56% of the total variance. The choice of considering three PC components was made to match our qualitative observations of the un-normalized firing patterns, and a broken stick model fitted on the cumulative explained variance of the first 30 PCs (Fig. 3*A*, right) confirmed that this was a reasonable threshold to separate the most informative components. The projection of the original data along the first three principal eigenvectors (Fig. 3*A*) identified the main neural patterns in our data. The task-normalized firing rates for the whole neural population were sorted according to their loadings on each of the first three PCs (Fig. 3*B*). The loadings on each of the first three PCs for pPys and pIns were compared using a Kolmogorov–Smirnov test (Fig. 3*C*).

Clustering of pyramidal neurons: in the PCA each cell receives a score (i.e., loading) for each PC, and therefore when classifying neurons based on a loading threshold it is possible for a neuron to be included in >1 class. The goal of clustering pyramidal neurons based on their loadings was to group neurons into one class only for analyses. For this, PCA was applied to the task-normalized firing rates from the 321 identified pPys. Collectively, the first three PCs explained 50.5% of the total variance and the respective loadings for each neuron were used as features in a *k*-means clustering algorithm. The optimal number of clusters was identified using the Akaike Measure of Information (Akaike, 1974) adapted for *k*-means algorithm (Goutte et al., 2001) and defined as follows:

$$AIC=ln\left(\hat{L}\right)-\left(KQ+1\right),$$

where the first term is the log-likelihood of the model (in our case, the specific clusters resulting from the *k*-means procedure), *K* is the number of clusters, and *Q* is the dimensionality (or number of features, 3 in our case). *K*-means algorithms can be considered as a form of expectation maximization algorithm for a Gaussian mixture model with equal weights and isotropic variances. The likelihood of our model was then estimated from the classification likelihood of each point *u_j_*, and it can be summarized in the equation:

L^=∏jN12πσ2⋅exp[−12(uj−ukj)σ2].

Where *N* is the total number of elements to classify, *σ^2^* is the average within-cluster variance calculated on all clusters, and *µ_kj_* is the centroid of cluster *k* to which *u_j_* is assigned. The Akaike information criterion (AIC) was calculated for values of *k* from 1 to 30 (Fig. 5*A1*), and a broken stick model was then used to select the number of clusters *K* = 4 that optimally balanced information and compression.

Familiar versus novel odor approaches: neural activities associated with approaches to familiar and novel odors were compared. Spike trains in a time interval of 4 s around each approach event (from −2 to 2 s) were binned in 40 intervals (0.1 s each). Events closer than 2 s to each other, to the end of delay marker, or to an error event were discarded. For each neuron, the firing rates obtained were normalized by the mean firing rate of the unit, and then averaged across all familiar approach events [mean familiar firing rate (fFR)], and across all novel approach events [mean novel firing rate (nFR)]. Neurons with a median number of spikes around the approach events <2 or with <6 trials available for both types of approaches were discarded, leaving *N* = 188 neurons available for the following analysis. Firing rates were smoothed using a moving average with a span of five bins. PCA was applied to the concatenated firing rate matrices (*N* × 80, where the first 40 columns contained the fFRs and the last 40 columns contained the nFRs). From the projections of firing rates along the first three PCs we obtained the trajectories and speeds of the whole neural population around both familiar and novel approaches in the PC space (Fig. 6*A*). For the following analysis we only included time bins up to 0.3 s after an approach. This was done to avoid contamination in the activity coming from either the dig or the reward; a correct dig happened before 0.3 s from a novel approach only in 1.8% of the trials considered (15 of 823). By concatenating the matrices vertically (2*N* × 23), a second PCA provided two loading coefficient sets for each neuron (1 for familiar and 1 for novel approaches). Firing rates for the positive and negative loaders on each PC for the two approaches were grouped and averaged (Fig. 6*B*). Distribution of absolute loadings on the first three PCs for the two approaches was compared by means of the Kolmogorov–Smirnov test (Fig. 6*C*). Incorrect choice trials: in 67 of the 77 sessions considered for analysis an incorrect choice trial was also recorded. Incorrect choices occurred when the animal dug into a non-novel odor bowl and it resulted in the session ending. Firing activity during a single incorrect trial was available for each of the 237 pyramidal neurons recorded from these sessions. Task-normalized firing rates for the incorrect trials were obtained between the four behavioral timestamps: Delay start; Delay end; Error dig; and End of trial and arranged in a matrix of size 237 × 100, where each row corresponded to the activity of a single neuron. For the same neurons, a random correct trial was selected for comparison, and the related task-normalized firing rates were arranged in a second matrix of size 237 × 100. The two matrices were concatenated row wise and PCA was applied to the resulting 237 × 200 matrix. PCA space trajectories and speeds of the neural population on correct and incorrect trials were then obtained from the projections of firing rate matrices along the first three PCs, which together explained 38.5% of the variance. Note that a single correct trial was selected for this procedure to keep the signal’s noise comparable in the two matrices. As a control for possible effects due to the trial’s order, we also tried to select the random correct trial among the last five correct trials. PCA space trajectories obtained in this case were similar to those obtained with the unconstrained selection of the random correct trial. Task-normalized firing rates for the 237 pPys were sorted according to their loadings on PC3 (Fig. 7*B*). Firing rates for the top 30% positive loaders on PC3 were compared in the two conditions (correct and incorrect trials) through a two-way ANOVA. The firing rates at specific times were compared via FDR-corrected rank sum tests, as described for Figure 2*C*. Similarly, neural activity trajectories during trial progression (Fig. 7*A*) for 125 pPys (from sessions with a span length of at least 9), were obtained by PCA on averaged pairs of consecutive trials (sliding window from 1 to 9, with a window length of 2).

## Results

### Task normalization and the classification of neurons

A timeline of the experiment is illustrated in Figure 1*A*. The OST, detailed in Figure 1*B*, is designed to assess working memory capacity in rodents (Dudchenko et al., 2000). For each experimental session, the number of novel odors correctly identified (span length) is a measure of memory performance. In this experiment, seven well trained rats were implanted with mPFC electrodes and participated in a total of 86 recording sessions (see Materials and Methods; Table 1). The span length distribution across all recording sessions (Fig. 1*C*) was found to be bimodal; unimodality was rejected using a calibrated version of Hartigan’s dip test (Cheng and Hall 1998; Ardid et al., 2015; Calibrated Hartigan’s dip test, *D*_(86)_ = 0.048, *p* = 7.2 × 10^−3^). The local minimum between the two peaks (span = 11.5) was then taken as threshold to separate “Low” and “High” span sessions. Nine sessions with span length smaller than five were excluded from all the following analysis because of the inadequate number of trials. Performance was uniform across the cohort of animals (Fig. 1*D*; ANOVA test, *F*_(6,79)_ = 1.78, *p* = 0.11) and across testing days (ANOVA test, *F*_(18,67)_ = 1.22, *p* = 0.27). Moreover, sessions labeled as High and Low span were equally distributed across recording days (categorical ANOVA test, *F*_(18,67)_ = 0.93, *p* = 0.55). Analyses of the rats’ choices during trials from which recordings were obtained demonstrate that as the number of bowls available increased, the probability of the rat choosing the novel (i.e., correct) bowl decreased and the total number of bowls visited before a choice was made increased (Fig. 1*E*). In addition, the average time between approaches to bowls did not change significantly as the number of bowls available increased (ANOVA test, *F*_(14,702)_ = 1.51, *p* = 0.1), whether the rat ultimately achieved a high or low span (Fig. 1*F*). Thus, experimenter cues or changes in locomotor activity are unlikely to have confounded analyses among sessions. Following pre-processing and spike sorting, we identified 382 single neurons active during the 77 sessions considered for analysis.

**Table 1.** Number of recording sessions and number of neurons recorded for each animal

Animal ID	No. of sessions	No. of sessions with span >4	No. of neurons
1	15	15	68
2	10	9	38
3	16	14	70
4	19	16	84
5	6	5	29
6	10	8	71
7	10	10	22

As each rat is permitted to forage at its own pace, the duration of foraging varies for each trail. To overcome this problem, we employed a targeted task-normalization of the firing rates. Figure 2*B2* shows a schematic of a single trial, where specific events were aligned to specific percentages of completion: the delay epoch covered from 0 to 70% of the trial, foraging epoch from 70 to 90%, and reward epoch (including the time to go back into the cage) from 90 to 100%. Through a time-normalized binning procedure (described in Materials and Methods) we obtained, for each neuron and each trial, a task-normalized firing rate trace spacing 100 bins, from the beginning to the completion of the task. For each neuron, the resulting firing rates were *z*-scored and then averaged across trials. The grand-average of normalized firing rates for the whole population of 382 neurons is shown in Figure 2*B1*.

Neurons were classified as pIn and pPy according to their average waveform. The waveform features considered were integrated into the first component of a PCA and two Gaussian models were fit on the distribution of first components (Fig. 2*C1*) to separate narrow waveforms (corresponding to pIns) and broad waveforms (corresponding to pPy). Details about the classification procedure are described in Materials and Methods. Average waveforms and mean firing rate distribution for the classified cells are shown in Figure 2*C2* and *C3*, respectively. At the end of the classification procedure 61 of the 382 (16%) were classified as pIns, whereas the remaining 321 were classified as pPy. Firing rates across the population of pIns were significantly different from firing rates across the population of pPys (two-way ANOVA, interaction cell class × time, *F*_(99,38000)_ = 3.02, *p* = 8.4 × 10^−22^), with significant differences (FDR-corrected rank sum, *p* < 0.05) during the first 13% of the task and during the whole foraging epoch (Fig. 2*D*). In particular, pIns exhibited a distinct pattern of activity, most prominently characterized by an increase in the average firing rate during the foraging and reward epochs compared with the delay epoch.

### Neural activity patterns robustly remapped between task epochs and predicted span

The most prominent neural activity patterns underlying the average firing rate profile were assessed via PCA. Figure 3*A* shows the first three PCs identified. The task-normalized firing rates for the whole population of neurons were sorted according to their loadings on each of the first three PCs (Fig. 3*B*). From the sorted firing rates, distinct neural populations were observed whose firing rates changed together throughout the different task epochs. In particular, groups of foraging-active neurons and delay-active neurons emerged when sorting according to the first PC (Fig. 3*B*, left); neurons active during the first part of the delay epoch (early delay) were separated from neurons active in the second part of the delay epoch (late delay) when sorting according to the second PC (Fig. 3*B*, middle); and neurons particularly active during the reward epoch were identified by the third PC (Fig. 3*B*, right). Remapping of neural activity signaled the transition between task epochs with a sharp and abrupt transition between delay and foraging epochs, and a smaller transition between foraging and reward epochs. Interestingly, pIns loaded more heavily than pPys on the first PC (Fig. 3*C*, left; Kolmogorov–Smirnov test: *D*_(321,61)_ =0.24, *p* = 4.9 × 10^−3^), indicating that interneurons mostly stayed active from the end of the delay and throughout the foraging epoch (in line with their average firing rate observed in Fig. 2*D*). No significant difference was observed for loadings on second and third PCs (Fig. 3*C*, middle and right; Kolmogorov–Smirnov test: *D*_(321,61)_ = 0.10, *p* = 0.63 for PC2; *D*_(321,61)_ = 0.12, *p* = 0.37).

**Figure 4.**
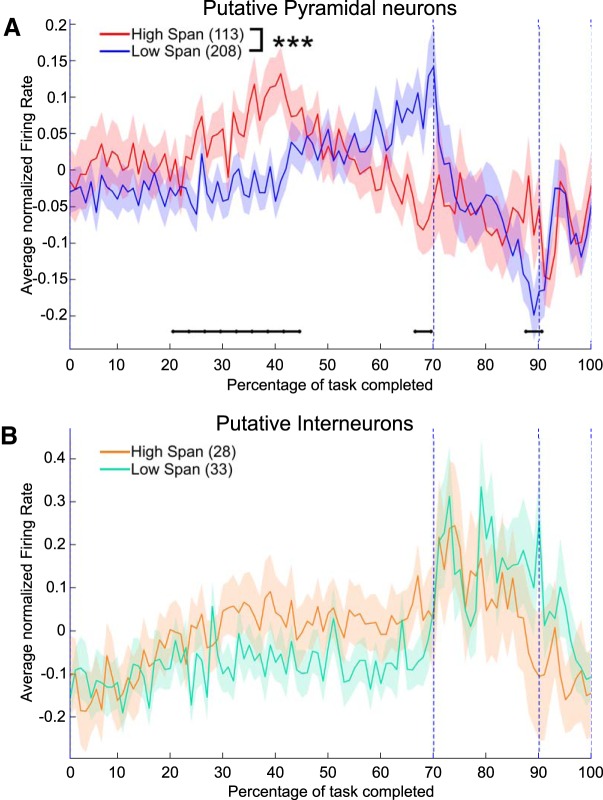
Activity of pyramidal neurons is predictive of span. ***A***, Grand-average (±SEM) of task-normalized firing rate for 321 pPys, separated according to the session’s span (low and high span were defined according to the threshold identified in Fig. 1*C*). Firing rates in the two groups were significantly different (two-way ANOVA, interaction between span class and time bin, *F*_(99,31900)_ = 1.72, p = 1.1 × 10^−5^). Black horizontal lines mark groups of time bins showing significant differences between low and high span groups (FDR-corrected rank sum, *p* < 0.05). ***B***, Grand-average (±SEM) of task-normalized firing rate for 61 pIns, separated according to the session’s span. Firing rates in the two groups were not significantly different (two-way ANOVA, interaction between span class and time bin *F*_(99,5900)_ = 0.87, *p* = 0.81). ***p < 0.001.

We then examined the relationship between neural firing rates and odor span. Sessions were separated into high and low span (according to the threshold defined in Fig. 1*C*) and firing rates were compared for both pPys and pIns in the two span groups. The average firing rates (±SEM) separated by span group are reported in Figure 4, *A* and *B*, for pPys and pIns, respectively. Firing rates of pPys recorded from low versus high span sessions were significantly different (two-way ANOVA, interaction span class × time, *F*_(99,31900)_ = 1.72, *p* = 1.1 × 10^−5^), with bin-by-bin differences observed especially during the middle part of the delay epoch (FDR-correct rank sum, *p* < 0.05). No significant differences were observed in the firing rates of pIns when comparing low and high span sessions (two-way ANOVA, *F*_(99,5900)_ = 0.87, *p* = 0.81).

**Figure 5.**
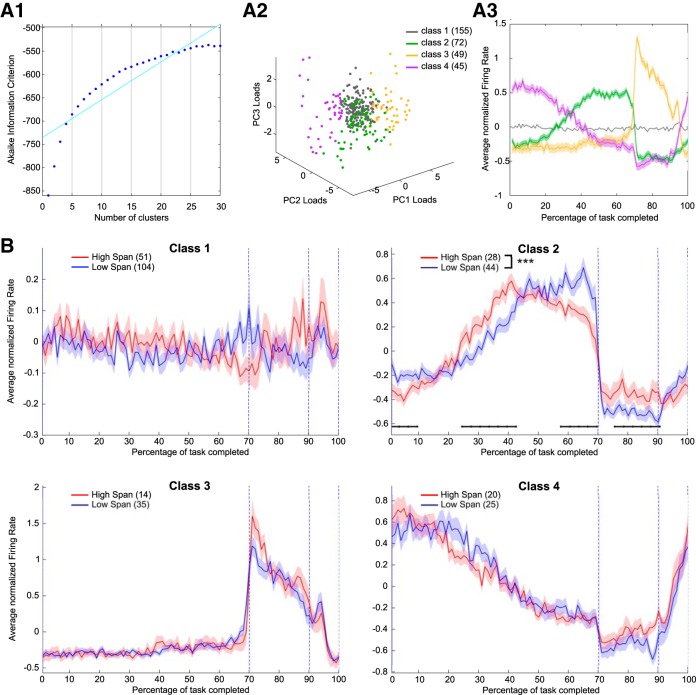
Identification of subpopulations of pyramidal neurons. ***A1***, AIC for the PCA-features *k*-means clustering, calculated for different number of clusters (*k*). The selected number of clusters (*k* = 4) was identified through a broken stick fit (cyan line). ***A2***, Loadings on the first 3 PCs for the population of 321 pPys clustered. Different colors indicate the different classes assigned. ***A3***, Average (±SEM) task-normalized firing rate for each of the classes identified. ***B***, Grand-average (±SEM) of task-normalized firing rate for each class of pPys, separated according to the session’s span (low or high). Only firing rates in Class 2 were significantly different (two-way ANOVA, interaction span class × time, *F*_(99,7000)_ = 3.59, *p* = 1.4 × 10^−29^). Black horizontal lines mark groups of time bins showing significant differences between low and high span groups (FDR-corrected rank sum, *p* < 0.05). No significant differences between firing rates in the low and high span sessions were observed in the remaining classes (two-way ANOVA, interaction span class × time: *F*_(99,15300)_ = 1.23, *p* = 0.06 for Class 1; *F*_(99,4700)_ = 0.93, *p* = 0.67 for Class 3; *F*_(99,4300)_ = 1.18, *p* = 0.11 for Class 4. ***p < 0.001.

Differences in neural activity patterns were observed in pPys across low and high spans. Because, as seen in Figure 3*B*, different neurons exhibit different firing patterns throughout the execution of the task, we asked whether specific subsets of neurons were responsible for driving the difference between low and high span seen in Figure 4*A*. We used a *k*-means clustering approach based on PCA-features (see Materials and Methods) to identify subclasses of pPys responding in a similar manner to the different task epochs. The ideal number of clusters (*k* = 4) was selected by means of the AIC (Fig. 5*A1*). The 3-D feature space considered for clustering (loading on the first 3 PCs for each neuron) and the classification results are shown in Figure 5*A2*. The average firing rate (±SEM) for each of the four classes identified is shown in Figure 5*A3*.

When looking at the relationship between span and firing rate for each of the subclasses identified (Fig. 5*B*), only Class 2 exhibited a significant difference between firing rates in the low versus the high span sessions (two-way ANOVA, interaction span class × time *F*_(99,7000)_ = 3.59, *p* = 1.4 × 10^−29^), localized both during the delay and the foraging epochs (FDR-correct rank sum, *p* < 0.05). Neurons in this class were increasing their activity during the second half of the delay epoch. In the high span sessions, such increases started earlier and decreased more gradually toward the end of the delay compared with the low span sessions. No significant differences between firing rates in the low and high span sessions were observed in the remaining classes (two-way ANOVA, interaction span class × time: *F*_(99,15300)_ = 1.23, *p* = 0.06 for Class 1; *F*_(99,4700)_ = 0.93, *p* = 0.67 for Class 3; *F*_(99,4300)_ = 1.18, *p* = 0.11 for Class 4).

### Neural activity changes upon approach to novel, but not familiar, odors

**Figure 6.**
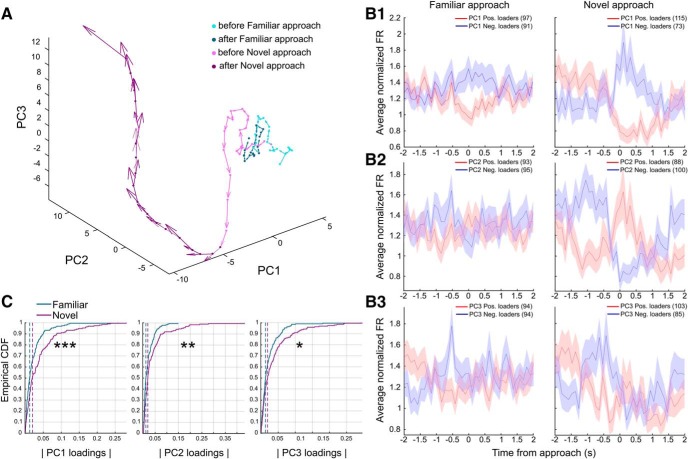
Distinct neural trajectories for familiar and novel odor approaches. ***A***, Neural activity trajectories in the PC space for 188 pyramidal neurons around familiar and novel approaches (time interval −2 to 2 s around each event, first 3 PCs explaining 56% of variance). Arrows indicate module and direction of trajectories’ speed. ***B1***–***B3***, Average normalized firing rates (±SEM) for positive and negative PC loaders for familiar approaches (left) and novel approaches (right). Loadings were obtained considering a time interval from −2 s to 0.3 s around each event. ***C***, Empirical cumulative distribution function (CDF) of absolute loadings on the first three PCs for familiar and novel approaches (time interval −2 s to 0.3 s around each event, first 3 PCs explaining 74% of variance). Absolute loading distributions in the two classes were different (Kolmogorov–Smirnov test: *D*_(188,188)_ = 0.22, *p* = 2.0 × 10^−4^ for PC1; *D*_(188,188)_ = 0.19, *p* = 2.5 × 10^−3^ for PC2; *D*_(188,188)_ = 0.15, *p* = 2.7 × 10^−2^ for PC3). *p < 0.05; **p < 0.01; ***p < 0.001.

During foraging, neural trajectories associated with the approach to familiar and novel odors were found to diverge right around the time of the approach (Fig. 6*A*). Trajectories were obtained through PCA on 188 neurons available (see Materials and Methods). Pronounced changes in neural activity were observed upon approach to a novel odor, while neural patterns associated with familiar odor approaches were weaker (Fig. 6*B*). The effect was statistically confirmed by comparing the distributions of absolute loadings on the first three PCs for familiar versus novel approaches (Fig. 6*C*; Kolmogorov–Smirnov test: *D*_(188,188)_ = 0.22, *p* = 2.0 × 10^−4^ for PC1; *D*_(188,188)_ = 0.19, *p* = 2.5 × 10^−3^ for PC2; *D*_(188,188)_ = 0.15, *p* = 2.7 × 10^−2^ for PC3). Note that both the pattern classification and the statistical comparison (Fig. 6*B*,*C*) were performed by including only the neural activity up to 0.3 s after the approach to avoid contamination in the activity coming from the following events.

**Figure 7.**
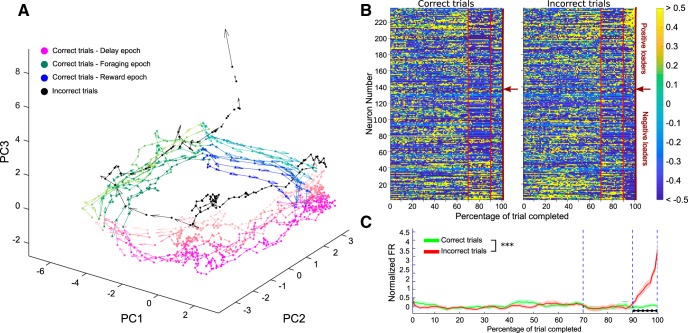
Divergence of the neural trajectory following an incorrect choice. ***A***, Neural activity trajectories in the PC space for 125 pPys during consecutive correct trials (1–9) and incorrect trials (black). Arrows indicate module and direction of trajectories’ speed. Different epochs of the task are color coded, transition between foraging and error epochs corresponds to a Correct dig for the correct trials and to an Error dig for the incorrect ones, trial progression is color-coded from darker to lighter. ***B***, Task-normalized firing rates for 237 pPys sorted according to their loadings on PC3 for correct (left) and incorrect (right) trials. PCA was performed on trial-normalized firing rates, and PC3 identified the error signal. Red vertical lines mark the End of the delay and the Dig event. Red arrows on the right side of each color-plot indicates the transition point between positive and negative loaders. ***C***, Grand-average (±SEM) of task-normalized firing rate for the top 30% positive loaders on PC3 (30 pPys) on correct and incorrect trials. Firing rates in the two groups were significantly different (two-way ANOVA, interaction between kind of trial and time bin, *F*_(99,5800)_ = 1.59, *p* = 2.0 × 10^−4^). Black horizontal markers indicate groups of time bins showing significant differences between correct and incorrect trials (FDR-corrected rank sum, *p* < 0.05). ***p < 0.001.

The divergences in neural dynamics observed in different aspects of the task (i.e., related to performance during the delay epoch and to the approach to novel odors during foraging) led us to speculate whether differences in mPFC neural activity could be detected on trials where the animal dug in an incorrect bowl. A PCA of correct and incorrect trials was performed to address this issue (see Materials and Methods). Figure 7*A* shows the neural trajectories for correct (color-coded by epoch) and incorrect (black) trials in PC space. The neural trajectories provide a qualitative assessment of the most predominant population activity patterns in mPFC that are observed across the delay, foraging, and reward epochs. For correct trials the neural activity patterns follow a circular trajectory in this space, where the neural activity patterns at the end of the trial are similar to those observed in the start of the trial, and no evident change appears as the session progresses. However, on an incorrect trial, a sharp divergence in the trajectory’s direction was observed along PC3 at the beginning of the reward epoch, which corresponded to the activity of a group of neurons that started firing during this time on error trials only (top positive loaders on PC3; Fig. 7*B*). Firing rates for the top 30% of positive loaders on PC3 (30 pPys) were significantly different on correct versus incorrect trials (two-way ANOVA, interaction between kind of trial and time bin, *F*_(99,5800)_ = 1.59, p = 2.0x10^−4^), with differences localized exclusively during the epoch following the dig (FDR-corrected rank sum, *p* < 0.05; Fig. 7*C*). Collectively, these data indicate that a unique signal emerges in mPFC immediately upon digging in an empty bowl that corresponds to a small subset of pyramidal neurons that start firing.

## Discussion

The present experiment is the first to directly measure patterns of neural activity during the OST. We assessed activity in mPFC given its established role in the OST and other tests of working memory. The main findings of the study are as follows: (1) span lengths were bimodal and longer spans were associated with differences in neural activity of putative pyramidal neurons during the delay; (2) sharp transitions in neural activity patterns emerge during the performance of the task that correspond to the onset of each behavioral epoch; (3) a transition was especially pronounced at the beginning of the foraging epoch where a group of putative interneurons were transiently and robustly active; (4) during foraging, neural activity patterns in putative pyramidal neurons were more robust during approach/digging of a novel than a familiar bowl; and (5) a group of putative pyramidal neurons becomes active following an incorrect choice. Collectively, these data highlight the rich and evolving dynamics in mPFC that emerge throughout the performance of the OST. Therefore, the contribution of the mPFC to the OST is likely broad and diverse and not limited to the maintenance of a working memory.

### Neural activity at the termination of the delay correlates with span capacity

Our analyses of mPFC neural firing during the OST revealed complex patterns of neural activity that evolved throughout each of the epochs. Neural activity was increased in a subpopulation of putative pyramidal neurons during the delay, which then decreased sharply at the beginning of the foraging epoch (Figs. 3, 5*B*). Similar increases in mPFC delay activity that also predicted task performance have been reported in other spatial WM tasks (Myroshnychenko et al., 2017). In the current study, neural recordings were acquired in well trained rats that likely anticipate the end of the delay; therefore, these changes in activity may reflect preparation for foraging. In addition, we observed that on high span trials, neural activity patterns at the end of the delay progressively became more similar to neural activity patterns observed during foraging (Figs. 4*A*, 5*B*). This phenomenon may provide a smooth transition of the network to the foraging state and possibly facilitate the maintenance of information across the transition of the delay to the foraging epoch.

Putative interneurons were identified by their extracellular waveforms and most predominantly positively loaded on PC1 (Figs. 2, 3). Interneurons exhibited a rapid and pronounced increase in activity during the transition from the delay epoch to foraging epoch. This increase in activity was commensurate with increases in activity of a subpopulation of putative pyramidal neurons during this time as well. However, this was the only time during the task when transient increases in interneuron activity were detected, thus raising the question of what types of computations they facilitate during the transition from the delay to foraging epochs. During the foraging epoch, animals are required to sample different odors and refrain from digging in ones previously encountered. Effective execution of this part of the task requires that a representation of the previously visited bowls be held online in memory. This is extremely memory intensive and requires that between 4 and 17 odors be accessible in memory. Retention of these items in memory would be facilitated by a neural code that is flexible (e.g., it can be readily activated and inactivated) and highly dimensional (e.g., has high capacity). Maintaining a tight balance between excitation and inhibition in cortical networks has been suggested to facilitate an efficient yet high capacity coding scheme (Denève and Machens, 2016). Therefore, we hypothesize that the concomitant increases in pyramidal and interneuron firing provide a network state that is capable of facilitating the transient maintenance of potentially large memory sets, such as those required to hold previously encountered odors in working memory. Although there is precedent for this idea (Harvey et al., 2012; Lim and Goldman 2013, 2014), a more direct test of this hypothesis will provide critical clues as to how mPFC flexibly adapts to meet the computational needs of a given behavior.

### Neural activity in mPFC signals approach to novel, but not familiar, odors

The OST also has elements of a test of novelty detection whereby responding must be inhibited to familiar odors and then initiated (i.e., a dig) whenever a novel odor is detected. This pattern of responding requires maintenance of “familiarity” for odors that have been experienced during the daily session and inhibition of digging when they are approached. We did not find evidence that a familiarity signal and/or inhibition of digging signal is maintained in mPFC on approaches to familiar odors. This was a surprising observation given reports of deficits in response inhibition following lesions of the mPFC (Miller and Cohen 2001; Fuster 2008; Chudasama 2011; Dalley et al., 2011). This suggests that errors in the OST following lesions of the mPFC may not be associated with computational processes required to inhibit behavior but rather incorrectly identifying an odor as novel.

Neural activity was strongly modulated during approach to a novel odor. This could be interpreted as a novelty signal which then triggers a dig in the correct bowl. The anterior cingulate cortex in humans/primates is proposed to code associations between rewards and actions, and in particular determine actions necessary to obtain rewards (Rushworth et al., 2011). Further, the mPFC in rodents may be involved in “working-with-memory” during WM tasks, a function that optimizes behavioral responding during these tasks (Horst and Laubach, 2012). As changes in neural activity were observed through approach, then digging, and retrieval of reward, they might also encode a more general signal that reflects the change in the behavioral requirements of the task during this epoch (e.g., stop foraging, dig, and retrieve reward).

Neural activity associated with approaches and initial digging on correct and incorrect trials did not differ. However, at the end of the digging, when the food pellet should be retrievable, robust differences in neural activity were observed. Upon receiving the food pellet on correct trials, neural activity patterns were qualitatively similar to those observed during the delay period. This is not surprising because the reward epoch signals the beginning of the delay. However, incorrect trials were uniquely characterized by a group of putative pyramidal neurons that increased firing when the animal would have been rewarded on a correct trial. It is possible that these neurons encode an “error” signal driven by the expectancy mismatch of expecting food and not receiving it. However, an incorrect dig also signaled the end of the task for that day. As recordings were performed in well trained animals, it is likely the animals understood this, and this signal may reflect environmental changes associated with the end of the task (e.g., being taken from the arena, etc.).

### Implications for theories of mPFC function during working memory and foraging

A number of neural activity patterns emerged throughout the OST. Robust transitions in the pattern of active neurons were observable across each behavioral epoch, which reflects a transiently stable population of (in)active neurons likely necessary to carry out the cognitive demands of each epoch. Similar phenomena have been observed in the mPFC of rodents engaged in foraging tasks (Lapish et al., 2008; Balaguer-Ballester et al., 2011) and operant tasks (Hyman et al., 2017) that require WM. These “metastable” states are thought to provide an important mechanism to organize activity across populations of neurons to optimize information processing (Balaguer-Ballester et al., 2018). In the current study, the transitions between these states may have facilitated the updating of action plans required between each behavioral epoch.

The OST has been proposed as one of the few tasks suitable for measuring WM capacity in rodents and therefore provides an opportunity for identifying the brain mechanisms that underlie WM. Given the impairments in WM capacity seen in numerous brain disorders, use of this the task may provide an opportunity to model WM deficits in rodents and develop novel treatment approaches. Indeed, the OST shares some features in common with span tasks used to measure WM capacity in humans. However, differences between the OST and other WM capacity tasks used in humans and primates are notable. For example, a long delay period (at least in the context of WM) exists between the addition of each novel bowl in a given session and rats achieve odor spans much higher than the typical WM capacity limits in humans or primates. These differences have led some authors to question the specific nature of the cognitive function(s) measured by the task (April et al., 2013; Dudchenko et al., 2013; Branch et al., 2014; Davies et al., 2017). This study highlights the diverse and evolving patterns of neural activity observed in the mPFC of rodents performing the OST. Future research assessing the necessity of the observed neural activity states for span will increase understanding of the mPFC’s contribution to cognition, including those operations required for WM tasks with a significant capacity component.

## Synthesis

Reviewing Editor: Mark Baxter, Mount Sinai School of Medicine Decisions are customarily a result of the Reviewing Editor and the peer reviewers coming together and discussing their recommendations until a consensus is reached. When revisions are invited, a fact-based synthesis statement explaining their decision and outlining what is needed to prepare a revision will be listed below. The following reviewer(s) agreed to reveal their identity: Mark Laubach.

This study provides some of the first data to illustrate how neuronal ensembles in the rodent mPFC vary in relation to the performance of a working memory task. Most published studies of mPFC ensembles have used simpler tasks, such as delayed alternation, which might not actually depend on working memory. The task allows rats to produce free-paced behaviours, in the course of which they must visit successively longer sequences of putative reward locations to identify the correct location of a buried reward by its novel odor, before digging to claim the reward. This task design offers both experimental benefits and challenges, in that the task sequence (delay, forage, reward) promotes a naturalistic foraging behaviour, offering opportunity to assess rich single-trial dynamics, increasingly appreciated to underlie complex frontal cortical-dependent processes (e.g. Afshar...Shenoy 2011, Stokes and Spaak 2016, Pandarinath...Sussillo 2018). Such processes are typically difficult to analyse on a trial-average basis (as performed in this manuscript) due to variation of trial durations, as well as the random (self-guided) sequence of cue sampling. The authors apply a time warping process to examine trial-averaged data before switching to event-triggered un-normalized data for the final two figures.

In general we were all pleased with the quality of the experimental design, data analysis, and scope of conclusions drawn from the paper, but there are some important concerns the authors should address in a revision. These are separated below into ‘major’ and ‘minor’ issues.

Major issues:

1) The stimuli used in the study are complex. It is possible that interactions of the experimenters with the stimuli and the animals during the behavioral testing influenced the animals' performance of the task, and also their neuronal activity. Video analysis, perhaps using machine learning methods, of the animals and the experimenters would be helpful to rule out any impact of the researcher on the performance of the animals and the neuronal activity. At a minimum, the authors may comment on aspects of their protocol designed to minimize influence of extraneous factors such as experimenter cues on task performance and neuronal activity.

2) The authors report that rats can successfully complete the task with a range of between 5 and 18 odor spans, performances across which are bimodally distributed across recording sessions. They state that the rats are trained to criterion pre-implantation but whether their performance is asymptotic during testing is not reported: They should report whether or not these ‘good days and bad days’ are randomly distributed across recording sessions, or whether there is a systematic trend in performance. Equally important, are the firing rates of recorded neurons stationary across the experiment duration, and are their modulations by running speed stationary across recording span sessions? If not then these effects could confound and should be accounted for in later analysis.

3) The authors use an established method to separate candidate populations of putative pyramidal and interneurons by recorded waveforms. They should show example histology to give a clearer depiction of where the wire arrays were located, since the schematics in Fig 1C-D are weakly informative. They should also acknowledge that the single units analysed are an overlapping population (since electrodes are fixed) and are presumably contributing to both low- and high- span sessions. Please also include spike isolation statistics.

4) To account for the varying trial durations between different spans the authors apply a time normalization protocol to the recorded single unit firing rates. Given the large range of odor spans reported this raises some major concerns that need to be addressed: On individual trials, the delay length is a constant 40s, but the foraging time is elastic to encompass visits to between 5-18 putative reward locations, and they only assign 20% of the normalization period to this variable span for the trial-averaged analyses (Figs 2-4). At the very least, they should provide some indication as to the trial durations that contribute to these analyses and ideally show examples of un-normalized trials (data not shown - line 459) and stratify them by duration, otherwise confidence in the trial-averaged results is restricted to the delay and reward periods.

5) The authors define three (weakly-)dominant Principal Components (collectively accounting for only 56% of the between trial variance, presumably - but not remarked upon - reflecting the complex trial to trial dynamics and variable trial durations recorded). They do not state how they choose this number of components or show examples of higher-order components, indeed the breakdown of variance explained by eigenvectors in Fig 3A shows the a modest effect of adding a third component, suggesting that the main result here is the distinction between foraging active and delay active neurons (the former strongly contributed to by loadings from putative interneurons), and that early- and late-delay classes of neurons should perhaps be summarised as one population.

6) The span-dependent changes in putative pyramidal firing rate reported in Fig4A are interesting but hard to interpret since the data for the high span sessions are contributed to by more trials, thus represent a summary of potentially many more observations than the low span sessions. The effects of reward certainty and confidence in encoded information are hard to disentangle. It would be beneficial to show trial averages across a common number of trials to account for this: for example, restricting the average to the first 1-7 trials from high-span against trials 1-7 from low-span sessions meeting this count. Equally, comparing (e.g.) trials 1-4 and 12-15 from within high-span sessions would highlight how the representations are changing with time/working memory load.

7) The time-aligned analyses of familiar/novel and correct/error signals reported in Figs 6-7 are well executed and begin to hint at the dynamics underlying the OST task. As above, the authors fall short of examining the progression of familiar/novel as the session progresses or stratify by the final session span. Some indication of the variance between trials would be beneficial here, to reflect confidence (Fig 6) and surprise at errors (Fig 7) - perhaps accompanied by behavioural statistics of whether the animals pause for different durations or deliberate/vacillate between bowls more as the task progresses, and the memory of more odors have to be evaluated against the most recently sampled.

Minor issues:

1) Justification for a statement in the Introduction: “Prior work has indicated that optimal working memory performance requires that ensembles of medial prefrontal cortex (mPFC) neurons track the various requirements of a task (e.g., epochs, rules) (Lapish et al. 2008, 43 2015; Durstewitz et al. 2010; Del Arco et al. 2017) which may facilitate performance monitoring and error detection in these networks (Hyman et al. 2017). Trial-specific information must also be maintained over a delay for optimal performance of WM tasks.”

To my knowledge, these studies were descriptive and did not use perturbation methods. The claims that they provide evidence that rodent WM ‘requires’ mPFC ensemble activity or that such activity ‘must be maintained’ should be rephrased.

2) The choice of colormaps in Figs 3 and 7. Please replace the plots in those figures that use the rainbow colormap with an alternative colormap that has uniform luminance, e.g. parula or viridis. See https://jakevdp.github.io/blog/2014/10/16/how-bad-is-your-colormap/

3) While the methods for data analysis were described in sufficient detail, I did not find mention of the actual software used for data analysis, e.g. Matlab?

4) Intriguingly, putative interneurons are reported to show a stronger task-dependent firing rate modulation than putative pyramidal cells. Alongside Figure 2C, I would be interested to see some measure of the within-session between-trial variance in the firing rates of each cell class (for example, the time-un-normalized Fano Factor for Pyr vs In firing rates associated the Delay/Reward periods or for successive familiar visits to putative reward locations). This would provide insight into the single trial dynamics of the circuit that is restricted to pyramidal neurons only in the later analyses, and as such would provide a bridge between the trial normalized and averaged data and the upcoming single-trial analyses.

5) The principal components reported could be considered as latent variables encoded by similarly tuned units: PC1 in Fig 3 shows a stronger contribution by positively-loaded putative Interneurons (pIn), but also contains a substantial fraction of pPyr. These data provide an important opportunity to test the extent to which these cells are functionally interacting within the group? Is there evidence from spike-spike cross-correlograms that the pIns strongly loaded in PC1 are receiving synaptic drive from pPyrs strongly loaded in the same PC? Similarly, are pIns loaded in PC1 actively and specifically providing synaptic inhibition to pPyrs in strongly loaded into PC2 but not PC1? This highlights the issue addressed in the later k-means clustering that neurons are loaded into multiple PC's thus would need a hard threshold for PC membership ratio applied for which I recommend using the categories specified by Fig 5. What about differential inhibitory tone during the Span-dependent differences in firing rate of Class 2 pPyrs reported in Fig 5B? Such an analysis would, I feel, strengthen the interpretation of the role of interneurons in controlling/gating the activity of cell assemblies.
